# VPS29 Is Not an Active Metallo-Phosphatase but Is a Rigid Scaffold Required for Retromer Interaction with Accessory Proteins

**DOI:** 10.1371/journal.pone.0020420

**Published:** 2011-05-24

**Authors:** James D. Swarbrick, Daniel J. Shaw, Sandeep Chhabra, Rajesh Ghai, Eugene Valkov, Suzanne J. Norwood, Matthew N. J. Seaman, Brett M. Collins

**Affiliations:** 1 Monash Institute of Pharmaceutical Sciences, Monash University, Parkville, Victoria, Australia; 2 Institute for Molecular Bioscience, The University of Queensland, St. Lucia, Queensland, Australia; 3 School of Chemistry and Molecular Bioscience, The University of Queensland, St. Lucia, Queensland, Australia; 4 Department of Clinical Biochemistry, Cambridge Institute for Medical Research, Cambridge University, Cambridge, United Kingdom; Institut Curie, France

## Abstract

VPS29 is a key component of the cargo-binding core complex of retromer, a protein assembly with diverse roles in transport of receptors within the endosomal system. VPS29 has a fold related to metal-binding phosphatases and mediates interactions between retromer and other regulatory proteins. In this study we examine the functional interactions of mammalian VPS29, using X-ray crystallography and NMR spectroscopy. We find that although VPS29 can coordinate metal ions Mn^2+^ and Zn^2+^ in both the putative active site and at other locations, the affinity for metals is low, and lack of activity in phosphatase assays using a putative peptide substrate support the conclusion that VPS29 is not a functional metalloenzyme. There is evidence that structural elements of VPS29 critical for binding the retromer subunit VPS35 may undergo both metal-dependent and independent conformational changes regulating complex formation, however studies using ITC and NMR residual dipolar coupling (RDC) measurements show that this is not the case. Finally, NMR chemical shift mapping indicates that VPS29 is able to associate with SNX1 via a conserved hydrophobic surface, but with a low affinity that suggests additional interactions will be required to stabilise the complex *in vivo*. Our conclusion is that VPS29 is a metal ion-independent, rigid scaffolding domain, which is essential but not sufficient for incorporation of retromer into functional endosomal transport assemblies.

## Introduction

Retromer is a protein complex peripherally associated with endosomal organelles, and controls trafficking of a number of critical cargo molecules within tubulovesicular carriers to the trans Golgi network (TGN) [1], [2], [3], [4]. Biochemical and genetic studies in yeast and higher eukaryotes have identified two distinct retromer sub-complexes; a core trimer composed of VPS35-VPS29-VPS26 (VPS: vacuolar protein sorting) and an associated homo or hetero-dimer of sorting nexin (SNX) proteins, containing combinations of SNX1, SNX2, SNX5 and SNX6. The current model postulates that the core complex is a cargo loading assembly that binds to the cytoplasmic tails of trafficking receptors such as the cation independent mannose-6-phosphate receptor (CI-MPR), Wntless, sortilin and DMT1 via the large VPS35 subunit [5], [6], [7], [8], [9], [10], [11], [12], [13], [14], [15]. The SNX proteins drive the membrane remodelling required to form the tubulovesicular transport structures [16], [17], [18], [19], and along with the small GTPase Rab7 may regulate recruitment of retromer to endosomal membranes through binding to phosphatidylinositol-3-phosphate (PtdIns(3)P) [18], [19], [20], [21].

The exact roles of the individual subunits of the core retromer complex remain unclear. The function of the large VPS35 subunit is perhaps the best defined, as it forms the central scaffold for assembly with VPS29 and VPS26 [22], [23], [24], [25], it binds directly to transmembrane cargo molecules [5], [7], [12], and also associates with SNX proteins [19], [23], [26]. It can therefore be thought of as the primary hub for the spatiotemporal assembly of functional transport intermediates. The roles of VPS29 and VPS26 are not as well understood. Each of these proteins is required for the stability of the core trimer *in vivo* [22], [24], [27], [28], [29], and based on structural similarity to arrestin molecules it has been suggested that VPS26 may play an ancillary role in recruiting cargo molecules or accessory proteins [22], [29], although there is currently no experimental evidence for this.

VPS29 is the smallest subunit of retromer and its structure reveals a striking similarity to Ser/Thr phosphatase enzymes [24], [27], [30]. This discovery led to the hypothesis that with VPS35 and VPS26, VPS29 may be the catalytic subunit of a trimeric phosphatase (core retromer), with some similarity to the PP2A holoenzyme [2]. Support for this came from the observation that VPS29 displayed weak phosphatase activity against phosphorylated peptides derived from the CI-MPR [31], although other studies could find no enzymatic function [24], [27]. Further evidence against the potential phosphatase activity of retromer came from the crystal structure of VPS29 bound to a fragment of VPS35 [24]. This revealed that the putative active site of VPS29 is buried within the interface of the two molecules, precluding access to potential phosphorylated substrates. The ability of VPS29 to function as a protein phosphatase therefore remains in question.

There are several notable features about the putative VPS29 active site and overlapping VPS35 binding interface. Firstly the central Asp side-chain, which bridges the two essential divalent cations in all other phosphatase enzymes, is altered to an Asn in VPS29 (Asn39). While it was found that Mn^2+^ could bind within this putative active site when soaked into crystals in high molar excess, this binding resulted in a significant rearrangement in the local structure, in particular affecting the orientation of Phe63 [27]. The Phe63 side-chain forms an intimate contact with VPS35 in the co-complex structure [24] and our modelling indicates that steric clashes could potentially inhibit binding to VPS35 if metal ions are present. Thus there are several possibilities regarding the role of metal binding by VPS29. It could be critical to phosphatase activity of VPS29, an activity that will either require other co-factors or conformational changes in the retromer complex, it may help to stabilise the VPS29 structure, it may regulate molecular interactions with VPS35, or it may play no functional role.

A second interesting observation is that the adjacent loop-helix region of VPS29 encompassing helix α3 is in a dramatically different orientation when the mouse crystal structure is compared either with apo or VPS35-bound human VPS29 despite no significant sequence differences [2], [24], [27], [30]. This raises the question as to whether this region may be able to adopt multiple conformations in solution before becoming ordered upon VPS35 association.

It was also shown that a hydrophobic surface on yeast Vps29p remote from the Vps35p-binding interface and putative active site is critical for association of the retromer core complex with the yeast SNX paralogues Vps5p-Vps17p, demonstrating a role for Vps29p in yeast retromer assembly [27]. Recently mammalian VPS29 was shown to be critical for interaction of the retromer complex with the Rab GTPase activating protein (GAP) TBC1D5, via the same conserved surface as demonstrated for the yeast Vps29p-Vps5p/Vps17p interaction [32]. As yet, direct binding of these proteins to VPS29 remain to be demonstrated.

In this study we use X-ray crystallography and NMR spectroscopy to examine the conformational dynamics and biomolecular interactions of VPS29. Specifically we address three related questions regarding the activity of VPS29 within retromer; (i) does VPS29 bind metals, what is their affinity for the protein, and do they have a functional importance, (ii) does VPS29 undergo conformational changes when incorporated into the retromer complex, and (iii) how does VPS29 function in coupling retromer to regulatory proteins such as SNX1 and TBC1D5? Our conclusion is that despite retaining vestigial metal-binding capacity VPS29 is in fact a metal-independent scaffolding protein, that does not undergo large scale conformational changes on retromer association and displays specific but weak binding to SNX1 in solution. We propose this interaction will aid in coupling the proteins during the formation of dynamic protein networks required for endosomal trafficking, but on its own does not lead to stable complex formation. Our study thus lends evidence to the theory that VPS29 has evolved from a precursor phosphatase enzyme as a regulator of protein-protein interactions but is no longer an active metalloenzyme.

## Results

### Experimental approach and NMR assignment of VPS29

**Figure 1:**
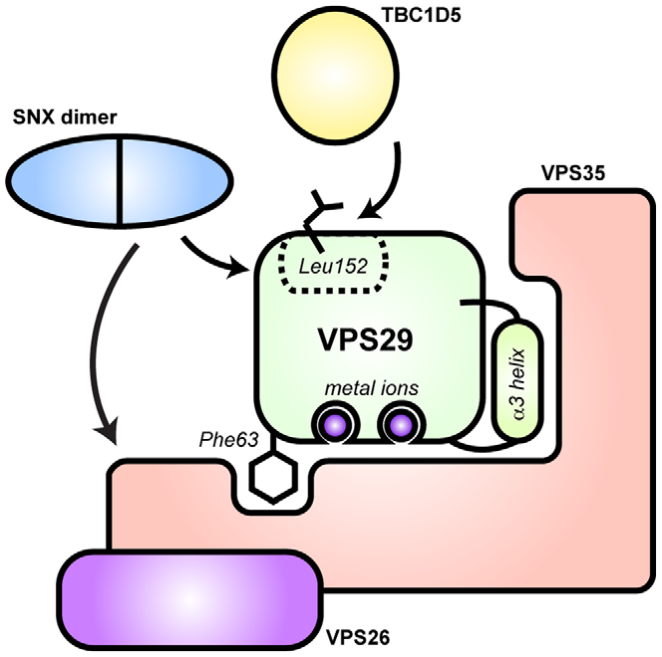
Interactions of VPS29 examined in this study. VPS29 has structural similarity to phosphatase enzymes and has the potential to bind two divalent cations [27]. VPS29 binds VPS35 with an interface that incorporates the metal-binding pocket, the α3 helix and the Phe63 side-chain [24]. The α3 helix is known to adopt different conformations in previous crystal structures [24], [27], [30], and Phe63 is known to adopt different conformations upon metal binding to VPS29 [27]. A conserved hydrophobic surface lies opposite to the VPS35 interface, and is known to be required for binding to TBC1D5 in mammalian cells [32], and to the heterodimeric SNX complex in yeast [27]. Critically mutation of Leu152 to Glu is known to abolish these interactions. Note, the SNX proteins are also thought to form contacts directly with VPS35 [20], [23].

The overall rationale of this study is outlined in **Fig. 1**. VPS29 has structural similarity to PPP Ser/Thr phosphatase enzymes and has a putative active site that has the potential to bind metal ions [27], [30]. VPS29 binds VPS35 with high affinity (*K*_d_∼250 nM), and the interface incorporates the metal-binding pocket, the α3 helix and the Phe63 side-chain [24], [25], [27]. The α3 helix is known to adopt different conformations in previous crystal structures [24], [27], [30], and Phe63 is known to adopt different conformations upon metal binding to VPS29 [27]. A conserved hydrophobic surface that includes the Leu152 side-chain lies opposite to the VPS35 interface. This is known to be required for binding to TBC1D5 in mammalian cells [32], and to the SNX complex in yeast cells [27]. Whether mammalian SNX homo and heterodimers use the same binding surface is unknown. From these previous studies, there are at least three possible, and not mutually exclusive, functions for the VPS29 protein. Firstly, it could be a metal-dependent phosphatase enzyme, secondly, it could be a scaffolding protein that utilises metal-binding in either a positive or negative regulatory capacity, or lastly, it may act as a metal-independent scaffold for assembly of retromer and functional endosomal trafficking complexes.

**Figure 2:**
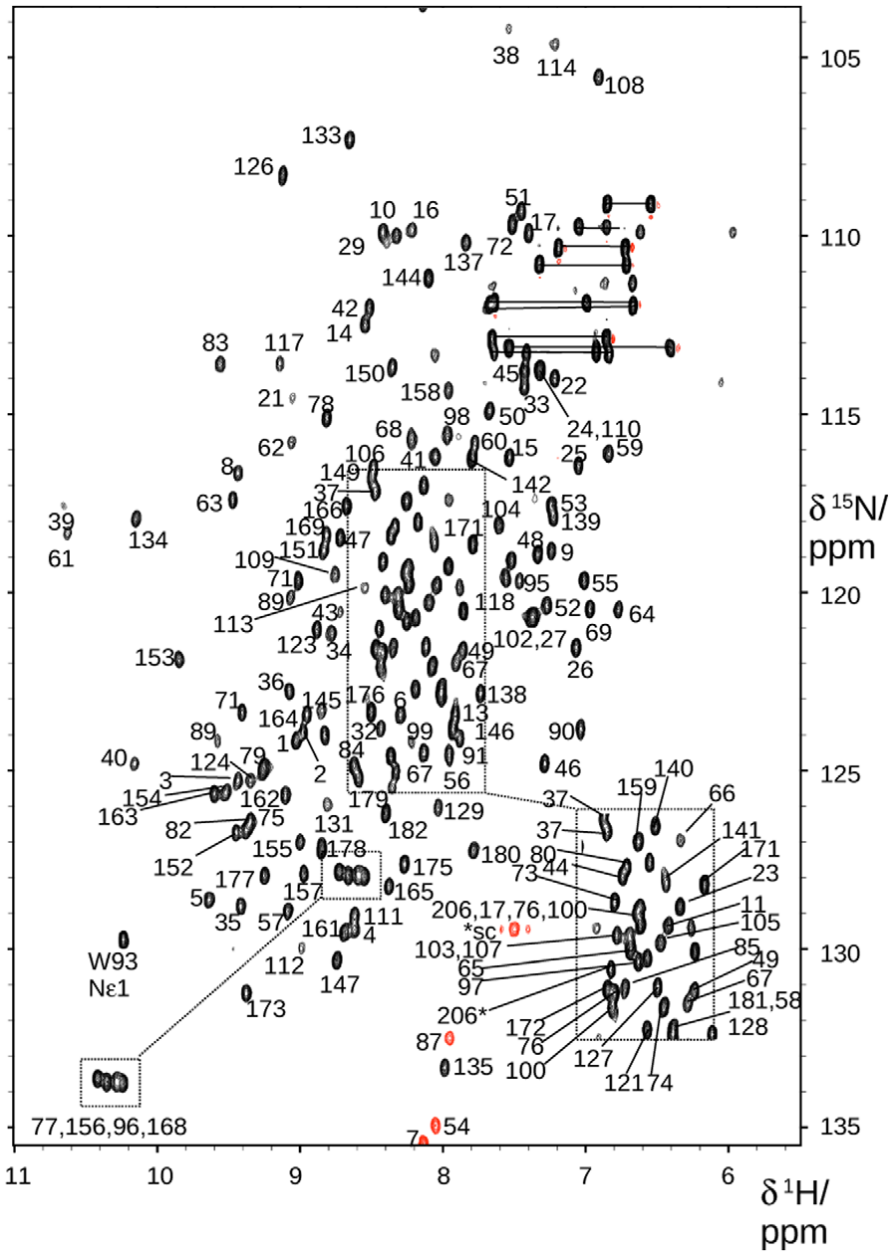
NMR assignement of VPS29. VPS29 [^1^H,^15^N]-HSQC spectrum. Assigned peaks are labelled by residue number.

In this study we use NMR spectroscopy and X-ray crystallography to examine the functional interactions of VPS29, their affinities, and potential conformational changes in VPS29 upon complex formation in order to distinguish between the above possibilities. As a prerequisite for ligand binding and structural experiments we performed ^15^N and ^13^C triple resonance experiments to assign the backbone ^1^H, ^15^N and ^13^C resonances of the mouse VPS29 protein (**Fig. 2**). VPS29 displays excellent NMR spectral properties, and we have been able to obtain 97% of the backbone assignments for the protein. Amide assignments are missing for Asn16, Ala20, Trp93, Gly94, Glu125 and Ile130. All backbone NOEs are consistent with the secondary structure observed in the X-ray structures of VPS29 (data not shown).

### Binding of VPS29 to Zn^2+^ measured by NMR spectroscopy

**Figure 3:**
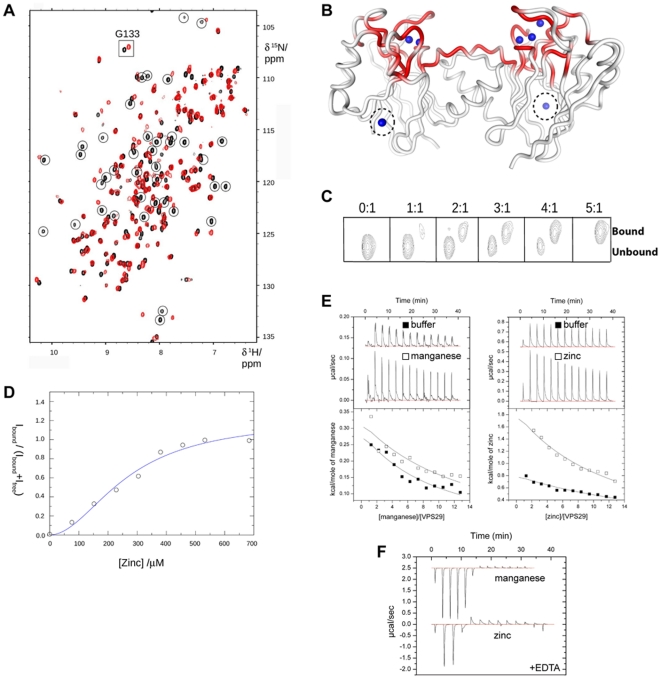
VPS29 binds Zn^2+^ in solution as determined by NMR spectroscopy. (**A**) A number of residues in the [^1^H,^15^N]-HSQC spectra of VPS29 show specific perturbations on addition of Zn^2+^. Spectra are shown of VPS29 in the apo state (black) or in the presence of 200 µM ZnCl_2_ (red). Resonances showing significant chemical shift changes are indicated. (**B**) Zn^2+^ binds to VPS29 in solution within the same major pocket identified by X-ray crystallography. Residues showing significant chemical shift upon Zn titration are highlighted on the structure in red (Δδ greater than 2 SD). Each monomer from the asymmetric unit of the Zn^2+^-bound mouse VPS29 crystal structure has been mapped, with crystallographically identified Zn^2+^ ions indicated in blue (see **Fig. 3** for details). Note however, that VPS29 is monomeric in solution, not dimeric. No significant evidence is seen for binding to the low occupancy Zn^2+^ site coordinated by Asp55, His57 and His33 (dashed circle). (**C**) Titration of VPS29 with Zn^2+^ can be followed by observing the change in intensity for the Gly133 NH resonance in bound and unbound states. (**D**) Plot of the bound and unbound intensity ratio for the Gly133 NH resonance as a function of Zn^2+^ concentration. The blue line shows the fit to the Hill equation. The estimated affinity is low with an overall *K*_d_>250 µM. (**E**) Binding of VPS29 to either Mn^2+^ or Zn^2+^ cannot be measured by ITC, confirming the low affinity of interaction. A weak endothermic signal is observed upon metal titration at 25°C but the binding affinity cannot be estimated. Top panels show raw data and bottom panels show integrated normalised data. No significant binding signals were observed under these conditions (0.04 mM protein, 2.5 mM metals, 25°C). Other metals including Mg^2+^, Ca^2+^ and Ni^2+^, and temperature regimes from 10–37°C produced similar negative results. (**F**) Mn^2+^ and Zn^2+^ bind to EDTA exothermically and with high affinity under identical conditions by ITC (0.2 mM EDTA, 2.5 mM metals, 25°C). The binding affinity is too high to be determined at the concentrations used.

We first revisited the binding of VPS29 to divalent metal ions to determine the affinity of the protein for these potential ligands. PPP phosphatases have a requirement for two divalent metal cations, typically transition metals Mn^2+^, Zn^2+^, Fe^2+^ or Ni^2+^, but not Ca^2+^ or Mg^2+^. We previously showed that VPS29 can bind to Mn^2+^, coordinating two metal ions using conserved Asp, Asn and His side chains in an almost identical arrangement to that seen in PPP phosphatase enzyme active sites [27]. To examine the affinity of metals with the VPS29 protein we employed NMR titration experiments. Binding of Mn^2+^ was not tested, as strong paramagnetic effects are expected to complicate data interpretation. Instead, binding of Zn^2+^ was followed by examining changes in the [^1^H,^15^N]-HSQC spectra of VPS29 upon addition of increasing concentrations of ZnCl_2_. These experiments indicated significant binding of the cation as assessed by the observation of widespread chemical shift perturbations (**Fig. 3A**) within either slow exchange or slow-intermediate exchange NMR time scales. Saturation occurred at approximately 500 µM Zn^2+^ (∼5-fold molar excess).

We assessed binding of Zn^2+^ to VPS29 by monitoring the intensity change of the free NH resonances, and those residues showing the greatest intensity change were mapped onto the VPS29 structure (**Fig. 3B**). The greatest alteration in the chemical environment corresponds to residues within the putative active site as identified from previous crystallographic experiments [27]. As discussed below there is crystallographic evidence for an additional Zn^2+^ binding site distal to the putative active site, however we do not find significant evidence of binding to this location by NMR experiments. As some protein precipitation occurred during the titration it was not generally possible to assign the resonances from the Zn^2+^-bound protein owing to sample instability. Nevertheless, the Gly133 HN resonance is well resolved and the assignment of the cation-bound form can be reliably inferred (**Fig. 3C**). Furthermore, as the distance to the metal-binding pocket is <9 Å, it is expected to be a reliable Zn^2+^ binding reporter for the active site of VPS29.

The affinity of metal binding was examined by plotting the intensity of the bound peak divided by the sum of the intensities of the bound and free peak for Gly133 against the molar ratio of zinc to protein. We observed a sigmoidal shaped binding curve, which is characteristic of cooperativity (**Fig. 3D**). This is not unexpected given that at least two cations are separated by only 3.5 Å within the putative active site (see below). Fitting the NMR binding data to the Hill equation suggests positive cooperativity (nH = 1.95) and an overall affinity (*K*_d_)>250 µM. No attempt to fit a multi-site, cooperative model to the NMR data was undertaken given the ambiguity in the number of metals in the binding site. This weak overall metal-binding affinity is consistent with isothermal titration calorimetry (ITC) experiments performed with VPS29 and a range of divalent cations. We were unable to detect significant association by ITC, indicating either no binding or affinities below *K*_d_ 200–300 µM (**Fig. 3E**). Binding to EDTA under identical conditions (**Fig.** 3F), and to a structurally related PPP phosphatase GpdQ [33] was readily measured (not shown), indicating that the inability to detect binding to VPS29 by ITC likely reflects a true low affinity association.

### Crystal structures of VPS29 bound to different divalent metals

**Figure 4:**
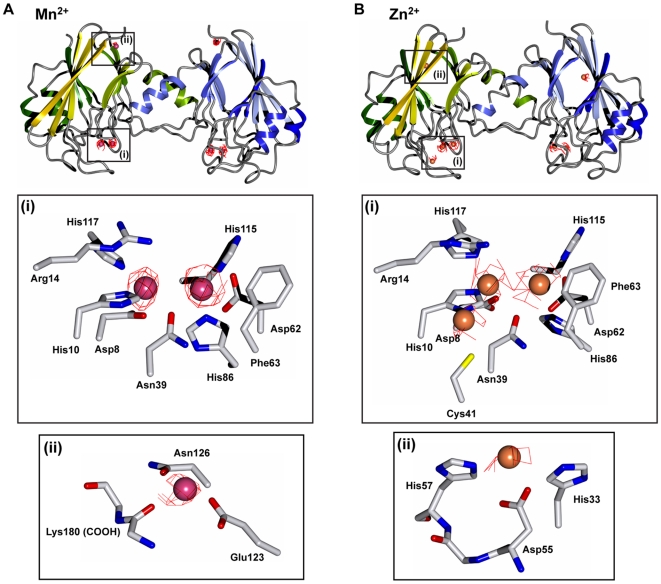
Crystal structures of VPS29 bound to Mn^2+^ and Zn^2+^. Crystal structure of VPS29 determined by X-ray crystallography bound to either Mn^2+^ (**A**) or Zn^2+^ (**B**). Top panels show overall protein structures as ribbon diagrams with anomalous difference maps contoured at 3σ shown in red. Each monomer from the asymmetric unit is indicated in green and blue. Mn^2+^ ions are shown as magenta spheres, and Zn^2+^ ions are shown as salmon spheres. Middle panels (i) show zoomed in regions of the putative active site residues and bound metal ions. Bottom panels (ii) show enlarged regions for minor low occupancy sites distal to the major binding pocket.

To examine the exact mechanism of VPS29 binding to Zn^2+^ we soaked protein crystals in a solution of ZnSO_4_ and determined its structure by X-ray crystallography (**Fig. 4**; **Table 1**). Under the same conditions, crystals could be placed in solutions containing MnSO_4_ at up to 100 mM concentration for several hours without noticeable effects on the crystal quality, however crystals cracked and degraded using as little as 5–10 mM ZnSO_4_ in minutes, thus limiting the resolution of the data we could collect. We obtained a 3.2 Å dataset of VPS29 crystals soaked in 2 mM ZnSO_4_ for 15 min, and importantly data was collected at the peak wavelength for Zn^2+^ to permit the exploitation of the anomalous signal in the identification of the metals in their cognate binding sites. A dataset was also collected for Mn^2+^-bound VPS29 at the same wavelength, where the expected anomalous signal is lower but still significant. Anomalous difference Fourier maps were computed using the Bijvoet differences collected at the Zn^2+^ peak wavelength and utilising the protein phases (**Fig. 4**). In the case of Mn^2+^ soaks, clear density is seen for two bound ions per protein chain, in an identical coordination to that observed previously [27]. The anomalous difference Fourier map shows clear density beyond 4σ over the background at the corresponding metal ion positions in both protein molecules of the asymmetric unit, and the f″/f′ values of 4 and 2 respectively at this wavelength are consistent with the sizes of the anomalous density peaks. In addition clear difference and anomalous difference density was observed for a bound ion coordinated by Glu123, Asp126 and the main-chain carbonyl of Lys180 in both chains, although density suggests this ion is at lower occupancy than the two found in the putative metal-binding pocket.

**Table 1:** Summary of crystallographic structure determination statistics^a^.

	VPS29 (Mn^2+^)	VPS29 (Zn^2+^)
**Data collection**		
Wavelength (Å)	1.2398	1.2398
Space group	P2_1_	P2_1_
Cell dimensions		
*a*, *b*, *c* (Å)	42.4, 69.1, 69.0	51.2, 68.6, 62.5
α, β, γ (°)	90, 105.4, 90	90, 105.7, 90
Resolution (Å)	19.61-2.40 (2.53-2.40)	19.77-3.20 (3.37-3.20)
*R*_merge_	0.071 (0.442)	0.106 (0.475)
*R*_meas_	0.092 (0.543)	0.134 (0.602)
*I*/σ*I*	12.8 (4.0)	10.2 (3.5)
Completeness (%)	99.7 (100)	99.5 (100)
Redundancy	4.5 (4.6)	5.1 (5.2)
Wilson plot *B* (Å)	40.8	89.3
**Refinement**		
Resolution (Å)	19.17-2.40 (2.58-2.40)	19.26-3.20 (4.0-3.2)
No. reflections/No. *R*_free_	14590/734 (2594/143)	6568/311 (2659/139)
*R*_work_/*R*_free_	0.197/0.243 (0.214/0.266)	0.185/0.242 (0.216/0.299)
No. Atoms		
Protein	2909	2899
Water	47	17
Metal ions	6 (Mn^2+^)	7 (Zn^2+^)
Average *B*-factor	44.2	76.7
R.m.s deviations		
Bond lengths (Å)	0.01	0.011
Bond angles (°)	1.15	1.37

a Highest resolution shell is shown in parentheses.

The coordination of Zn^2+^ within the metal-binding pocket is essentially identical to Mn^2+^, confirming that the ability to bind metals within this pocket is a general feature of the protein. A pair of Zn^2+^ cations are coordinated by the His and Asp side chains of the central pocket, and bridged by the conserved Asn39 side chain. Intriguingly, we also observe an additional bound Zn^2+^ ion in one of the two VPS29 monomers (chain A) in the asymmetric unit. This Zn^2+^ ion is coordinated by His10 and Cys41, in very close proximity to the active site. The lack of a similar bound Zn^2+^ ion in chain B is likely due to an inhibitory crystal contact in this region. Evidence for a more weakly occupied Zn^2+^ ion is also found at a distal site coordinated by Asp55, His57 and His33 of each chain. This latter, apparently lower affinity site was not detected in our NMR titration experiments (see above).

### VPS29 and retromer do not have phosphatase activity against CI-MPR

**Figure 5:**
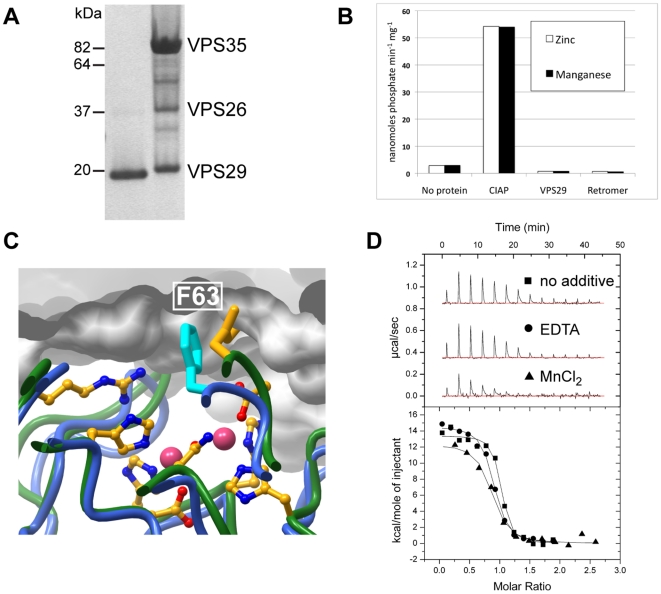
Metals do not affect VPS29 phosphatase activity or interaction with VPS35. (**A**) SDS-PAGE gel showing purified VPS29 and trimeric retromer proteins used for phosphatase assays stained with Coomassie Blue. (**B**) No detectable phosphatase activity was measured for VPS29 alone or in complex with VPS35 and VPS26. Phosphatase assays used the CI-MPR peptide CSSTKLVSFHDD(pS)DEDLLHI. The release of phosphate was measured using Biomol Green reagent and colorimetric assay at 620 nm. Calf intestinal alkaline phosphatase (CIAP) is shown for comparison. (**C**) When VPS29 has bound metal, the conformation of Phe63 is altered such that it may clash with VPS35 and inhibit binding. The diagram shows a close up of the interaction between VPS29 and VPS35 [24]. The Mn^2+^-bound VPS29 structure (green ribbon, and yellow side-chains) is overlayed with VPS35-bound VPS29 (blue ribbon and cyan side-chain). VPS35 is shown in surface representation. (**D**) No significant difference is observed in binding to VPS35 in the presence of EDTA or MnCl_2_ indicating that metals do not influence complex formation. VPS29 interaction with VPS35 was analysed by ITC; top panels show raw data and bottom panels show integrated normalised data.

Our NMR and crystallography experiments confirm that VPS29 can associate with different divalent metal cations, but the very low affinity of these metals calls into question the functional importance of their binding. As PPP phosphatase enzymes have an absolute requirement for divalent metal cations for activity, the key question is whether VPS29 is able to function as a metallophosphatase. As outlined above we have not detected activity using small molecule substrates [27], but it has been reported that VPS29 has activity against a phosphorylated peptide derived from the cargo protein CI-MPR, and this can be enhanced by the presence of other retromer subunits [31]. However, like Hierro and colleagues [24], when we perform similar experiments with purified proteins we do not observe any phosphatase activity towards the phosphorylated CI-MPR peptide (**Fig. 5A, 5B**). Data is shown only for experiments incorporating Zn^2+^ and Mn^2+^. Other ions, different pH and higher protein concentrations yield similar results. Our data, and that of Hierro and colleagues [24], strongly argues therefore that VPS29 is not a metal-dependent phosphatase enzyme.

### VPS29 binding to VPS35 is not affected by metal ions

Apart from promoting enzymatic activity, another potential role for metal-binding by VPS29 may be to regulate molecular interactions via conformational changes in the protein. Crystal structures of VPS29 bound to Zn^2+^ or Mn^2+^ presented here and previously [27] show that the presence of metals within the conserved pocket causes a small but marked rearrangement of residues, resulting in particular in a significant shift of the Phe63 side chain orientiation. Within the VPS29-VPS35 sub-assembly of retromer, this Phe63 side chain is tightly buried [24], and our modelling suggests that metal-induced conformational change could alter the normal association of VPS29 with the VPS35 subunit by steric interference (**Fig. 5C**). Our previous pull-down experiments do not support this [27], and neither do ITC experiments performed in the presence and absence of excess MnCl_2_ or EDTA that show no significant change in the affinity or thermodynamics of VPS35 association (**Fig. 5D**). Similar experiments could not be performed using ZnCl_2_ due to rapid precipitation of VPS35 in the presence of this salt even at concentrations of as little as 0.5 mM.

### Structural comparison of VPS29 in crystalline and solution environments

The NMR assignment of VPS29 places us in an ideal position to assess conformational dynamics of VPS29 in solution, and potential structural changes in the protein. From a comparison of three deposited structures of VPS29 (human VPS29, PDB 1W24; human VPS29 in complex with VPS35, PDB 2R17; mouse VPS29, PDB 1Z2X) distinct structural differences are observed. Superimposing the apo and VPS35-bound human protein yields a low r.m.s.d. of 0.54 Å over 178 backbone atom pairs. Subtle differences are observed in the loop residues 140–145, and there is also missing density for the N terminal loop of helix α3 (residues 93 and 94) in the apo human VPS29 structure [30]. In comparison, a superposition of mouse VPS29 and either human structure demonstrates a dramatic difference in the orientation of helix α3 (residues 96–106) of ∼83°, which would preclude binding to VPS35. We have investigated the functional relevance of these observed structural differences in the context of potential inter-subunit crystal packing anomalies and/or as possible effects of dynamics in solution. In this, we determined whether the interacting helix has significant mobility in the isolated, monomeric VPS29 and if not, whether a substantial reorientation is required in solution to bind VPS35. To this end, we measured ^15^N relaxation parameters to survey the dynamics of VPS29 on the ps-ns timescale, and have also determined the orientation of helix α3 in solution by NMR using primarily Residual Dipolar Couplings (RDCs) combined with limited NOE data.

### Structural dynamics of VPS29 in solution

**Figure 6:**
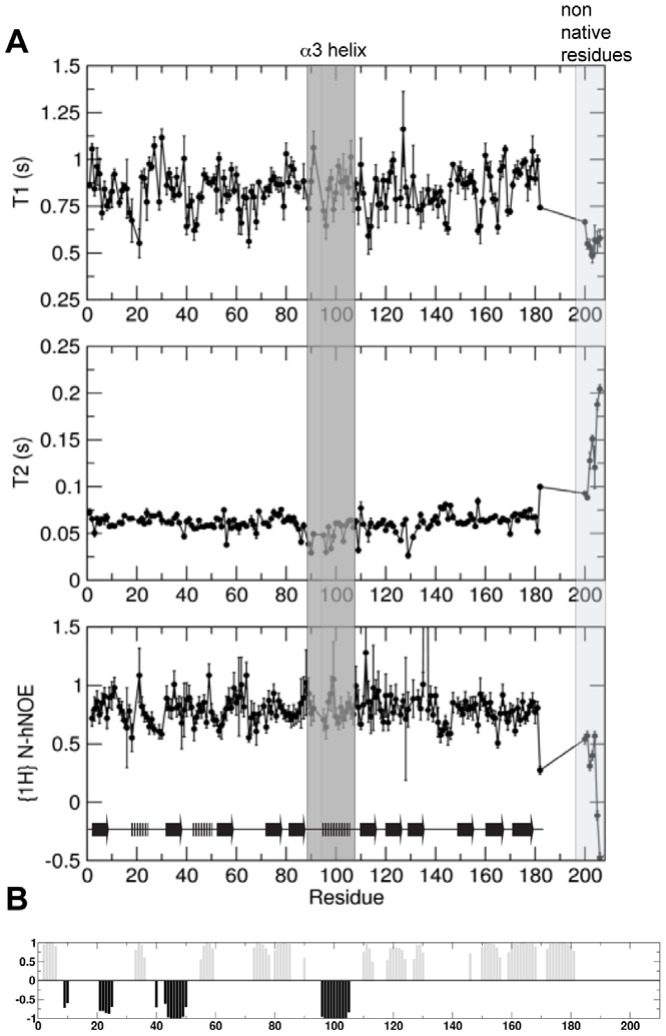
^15^N NMR relaxation data for VPS29 indicates a generally rigid structure with no large-scale mobility with well defined secondary structural elements. (**A**) Longitudinal T1 and transverse T2 relaxation times as well as the {^1^H}^15^N heteronuclear NOEs are shown as a function of protein sequence. Residues within helix α3 are highlighted in grey. Six N-terminal (non-native) resides are shown and are labelled as residues 201–206. The protein secondary structure is indicated at the bottom of the figure. Data was recorded using a 600 MHz spectrometer. (**B**) The TALOS+ artificial neural network (ANN)-predicted secondary structural elements of VPS29. Length of bars corresponds to probability of a residue to be helix (black) or β-strand (grey).

To investigate the dynamic properties of VPS29 we recorded ^15^N T1, T2 and a ^15^N heteronuclear NOE experiment at a single field strength (600 MHz) (**Fig. 6**). A ratio of the T1 and T2 values is in close agreement with that predicted using HYDRONMR [34], consistent with a monomeric VPS29 in solution (with a correlation time of 11.1 ns), and agreeing with gel filtration data. On first inspection, the relaxation data indicates that VPS29 has no particularly large amplitude mobile segments as would be indicated by a stretch of low ^15^N NOE values<0.5 (**Fig. 6**). Amide resonances corresponding to the N-terminal non-native residues derived from the expression vector do show the low, and typically negative ^15^N NOEs characteristic of a disordered peptide. Several residues however, show a significantly reduced ^15^N NOE value of ∼0.5–0.6 (Leu18, Gln65, and Asn140 through to Asn145) and are found in loop regions. These residues are buried in the interface of the VPS29-VPS35 complex [24], and their relative fast timescale mobility in the apo VPS29 protein is consistent with our previous hypothesis that this region undergoes structural rigidification upon complex formation [25]. The sidechain of Leu142 is seen to change conformation significantly between the apo state and in the complex with VPS35. The conformational change is accompanied by a ∼9 Å translation in the sidechain δC methyl positions of Leu142. Further evidence to suggest this region is mobile in apo VPS29 is that a comparison of the RDC data (see next section) to any of the VPS29 structures for this region gave a poor fit, as expected for a mobile segment.

Backbone torsional angle and secondary structure prediction using ^13^CA, ^13^CB, ^13^C, ^15^N and ^1^HN chemical shifts within TALOS+ [35] showed that the secondary structural elements of VPS29, including the α3 helix, are predicted with high confidence in solution to be the same as in the crystal structure (**Fig. 6B**). Furthermore, the ^15^N NOE data shows that the α3 helix and preceding loop region appears not to have large amplitude mobility in solution on the fast (ps-ns) timescale. These finding prompted us to look more closely at the relative orientations of the structural elements of VPS29, and consider using RDC measurements to specifically investigate the conformational orientation of the α3 helix in solution as described in the following section. Careful inspection reveals a noticeable decrease in the T2 values for a few, but not all residues in the α3 helix and preceding loop (residues 90, 96, 98, 103). This observation is suggestive of either R_ex_ contribution to the ^15^N T2 for these residues, or that the helix is possibly pointing along the main axis of the diffusion tensor, and these residues are tumbling effectively like that of a larger protein. Given that amide broadening is not observed from residues along the length of the helix (data not shown), it is more likely to be due to the indirect effect of e.g. proximal sidechains rather than µs-ms motion of the helix in general.

A number of residues show decreased T2 values, paralleled by the observation of significant broadening in the ^15^N-HSQC spectra, indicating µs-ms motions (**Fig. 6B**). These are Asn39, Val56, His86, Gly94, Asp109, and Tyr129. Asn39 (the central bridging residue of the metal-binding pocket) is in a loop region and the observed T2 and broadening is most likely due to general motion in this loop region. Val56 HN is at the edge of the sheet and although several backbone long range NOEs are observed between residues 33/55, 34/57 and 34/56 confirming the parallel sheet arrangement, the HN is solvent exposed and not directly part of the sheet hydrogen bond network that would presumably dampen any motions. Interestingly, Tyr129 and Ile112, which are just visible in the HSQC and gave substandard exponential decays for T1 and T2 fitting, lie underneath the α3 helix in the middle of the sheet. Broadening may be due to motions not in loops but from residues in the proximal helix α3, the side-chains therein or simply from nearby residues.

Some amide resonances are broadened beyond detection in the ^15^N-HSQC experiment which suggests severe µs-ms conformational exchange for these amide residues (or the possibility that they resonate directly under the water). These include Trp93 and Gly94. Interestingly, the parallel lack of density for this region in the human X-ray VPS29 structure is also indicative of conformational exchange and flexibility. It has to be noted, that the observation of line broadening in NMR spectra does not imply that the populations of the other conformations in exchange are large, as populations as little as 1% can lead to substantial broadening [36].

### Solution conformation of the α3 helix of VPS29

**Figure 7:**
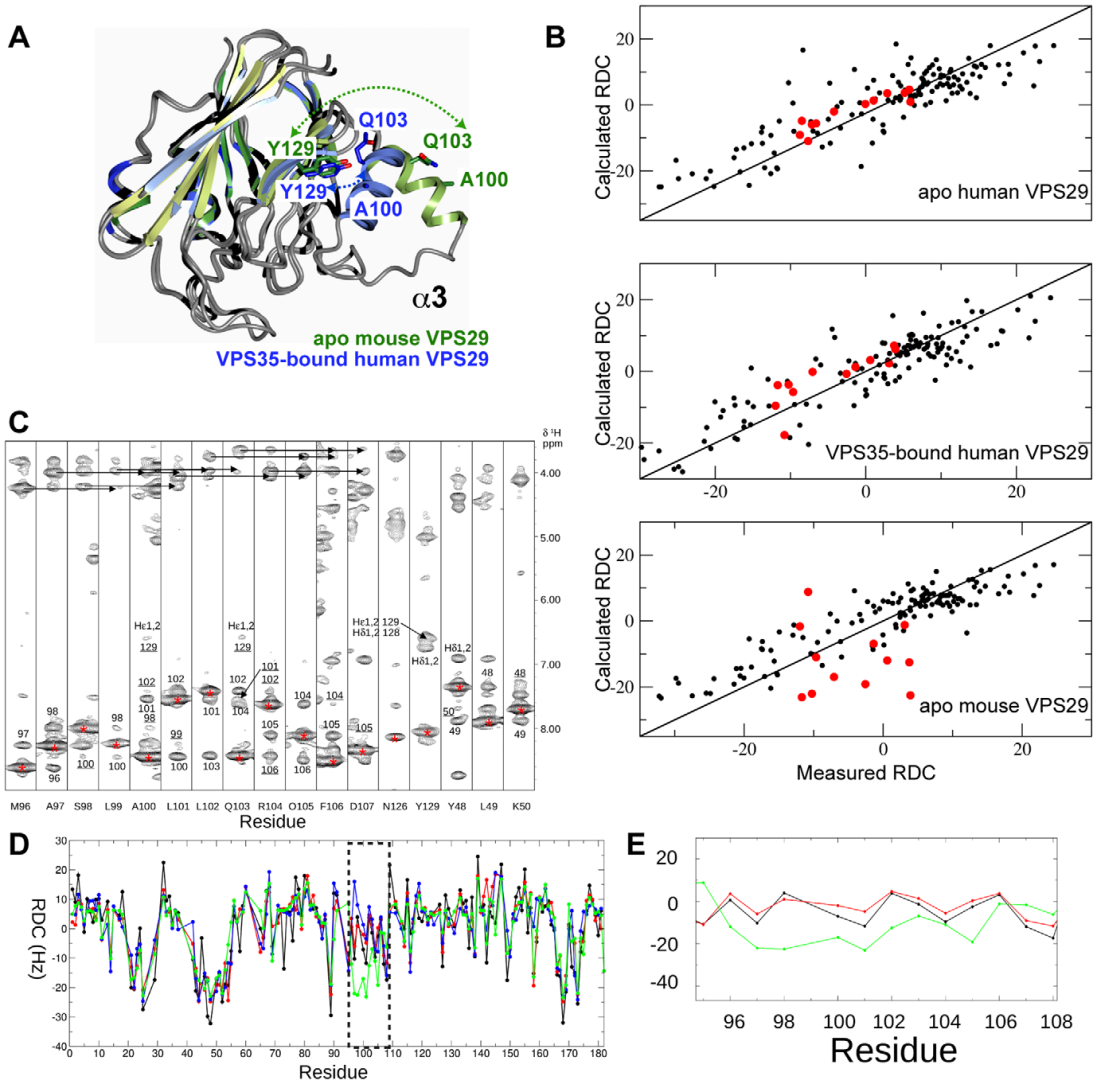
The α3 helix of VPS29 adopts a compact conformation in solution. (**A**) Comparison of previous VPS29 crystal structures reveals differences in the orientations of the α3 helix. VPS29 adopts an extended α3 orientation in the mouse apo VPS29 crystal structure (PDB 1Z2X; green), and a compact orientation in the VPS35-bound human VPS29 structure (PDB 2R17; blue). Ala100, Gln103 and Tyr129 are shown for each structure, to show residues for which long-range NOEs are observed. In the apo mouse VPS29 structure these residues would be too far apart to observe these NOE contacts. (**B**) RDC correlation plots indicate the apo mouse VPS29 protein adopts a compact structure in solution, where the α3 helix is similar to the VPS35-bound conformation, but not the previous mouse VPS29 crystal structure. Shown are 133 ^1^D_HN_ RDCs fitted to the apo human VPS29 structure (PDB 1W24), human VPS29 in complex with VPS35 (PDB 2R17) and the mouse VPS29 structure (PDB 1Z2X). The residues of helix α3 are highlighted in red (95–107). Residue 99 was not included due to severe overlap in the 2D ^15^N IPAP spectra. Single value decomposition analysis of the HN-N RDCs to the apo human, VPS35-bound human or apo mouse X-ray structures yielded the following: The largest component of the alignment tensor (Szz) were 1.31e^−3^, 1.35e^−3^, 1.09e^−3^ and Rhombicities (S_yy_−S_xy_/S_zz_) were 0.53, 0.53 and 0.48. (**C**) 2D ^1^HN-^1^H strips from the 3D ^15^N NOESY-HSQC show the NOE connectivities along helix α3 (residues 97–107). Diagonal peaks for each strip are labelled with an asterisk and proximal protons that give rise to observable NOEs are annotated. Medium range i, i+2 ^1^HN-^1^HN NOES are underlined while medium range i, i+3 and i, i+4 ^1^Hα-^1^HN NOES are identified with arrows. Both are diagnostic of a regular alpha helix. Two long range NOEs to the Hε protons of Tyr129 from the ^1^HN of Ala100 and Gln103 are labelled as well as the long-range (cross sheet) ^1^HN-^1^HN NOE between Y129 and F122. (**D**) ^1^D_HN_ RDCs observed (black) compared to those calculated for each residue for mouse VPS29 structure (PDB 1Z2X, green), apo human VPS29 structure (PDB 1W24, red) and human VPS29 in complex with VPS35 (PDB 2R17, blue). (**E**) Detail comparing the close fit of the ^1^D_HN_ RDCs to the α3 orientation in the apo human VPS29 compared to the poor fit to the mouse α3 orientation.

As described above, previous crystal structures show that VPS29 can adopt two very different conformations, an open form where the α3 helix is free of intra-molecular contacts forming an extended structure, and a compact form whereby the α3 helix is packed closely against the central β-sandwich of VPS29 (**Fig. 7A**). The compact form is observed in the crystal structure of apo human VPS29, and the crystal structure of human VPS29 bound to VPS35(476–780) where helix α3 also forms extensive contacts with the VPS35 protein [23], [28]. The extended form of VPS29 is observed in the crystal structure of mouse VPS29, and in this structure the open α3 helix is stabilised by contacts that create a non-crystallographic VPS29 dimer within the asymmetric unit [27]. In the extended conformation observed in mouse VPS29, helix α3 would preclude complex formation with VPS35. As the human and mouse proteins are essentially identical in sequence, the observed conformational differences are either due to crystal packing or reflect a true dynamic flexibility in a region of VPS29 critical for VPS35 binding (or both).

Over the last decade there has been a growing interest in using RDC calculations as a powerful additional parameter for the structure refinement of proteins, DNA and RNA and when high-resolution structures are available, in the rapid determination and validation of domain-domain orientations in macromolecular complexes [37], [38]. More recently, their utility has been central to the determination of protein structure using backbone only data along with Rosetta modelling methodology [39]. RDC measurements have also been elegantly correlated with dynamics in conjunction with relaxation measurements [40]. Herein we use RDC measurements to validate the orientation of the α3 helix in solution.

RDCs were obtained in a PEG/hexanol alignment media. These measurements allowed us to derive the alignment tensor by single value decomposition within PALES by fitting 133 measured ^15^N RDCs [41] to the X-ray structures of mouse or human VPS29 and to back calculate the RDCs for each residue (**Fig. 7B, D, E**). From the PALES calculations, the correlation between the 16 observed and calculated RDCs for the α3 helix region (residues 88–104) are significantly better when fitted to either of the human VPS29 structures than to the mouse VPS29 protein. The correlation (R) for the 16 RDCs fitted to the mouse X-ray structure is very poor (0.17) compared to that when fitted to the X-ray structure of the human apo (0.85) or human/VPS35 structure (0.89). For the α3 helix region, the measured RDCs are in excellent agreement with those calculated from the human structure (**Fig. 7D, E**) and slightly less so for the human/VPS35 structure. Inclusion of the RDCs for the 142–146 loop were not included in the calculation of the orientation of the α3 helix (although they did not skew the final result) but deviations to the measured RDC of up to 28 Hz were observed (**Fig. 7D**). It is clear from the relaxation data (see previous section) that this region is mobile in solution and different conformations are observed in the X-ray structures. We also conclude that the difference between the X-ray and solution structures is not due to interaction with the neutral PEG/hexanol medium as there were minimal chemical shift changes. The reduction in the ^15^N T2 values for selected amides along the helix does not contradict the RDC findings which show the major helix conformation is very close to that of the human crystal structure and therefore any motion (reflected in a reduced ^15^N T2) is minimal or an indirect effect.

Adopting a classical NOE analysis to understand the orientation of the α3 helix is problematic as the NOE distance restraints are predominantly short range for this scaffold. In this situation, the orientation of the helix is derived largely by the calibration of the NOEs in the loop regions either side of the helix and the observation of a few long-range restraints if they exist to the helix itself. Compounding the problems with this style of analysis is that the amides from the residues in the N-terminal loop of the helix are significantly broadened (Gly94) or absent (Trp93) in the ^15^N-HSQC spectra as described above, reducing the number of potential NOEs in this connecting region. Nonetheless, NOE analyses provided useful corroborating evidence for the findings using RDC correlations. Firstly, evidence that helix α3 is not mobile in solution comes from strong sequential HN-HN NOEs and several medium range HN-HN(i, i+2), Hα-HN(i, i+3) and Hα-HN(i, i+4) NOEs observed along the helix (**Fig. 7C**), diagnostic of a regular rather than frayed structure. Furthermore the residues in the helix are not substantially broadened in the ^15^N-HSQC spectrum as might be expected if the helix is undergoing large amplitude (ms-ms) motions, often observed in enzyme active sites for example [42]. Finally classical NOE evidence further suggests a predominance of the close-packed orientation of helix α3 in solution by the observation of two long range NOEs between Ala100HN, Gln103HN and the side-chain Hε protons of Tyr 129. The distances between these two proton pairs in the open α3 orientation is far too long (18 and 13 Å respectively), but in the compact conformation are short enough for detection by the NOE (3.87–4.01 Å) (**Fig. 7A**).

### Interaction of VPS29 with regulatory proteins

**Figure 8:**
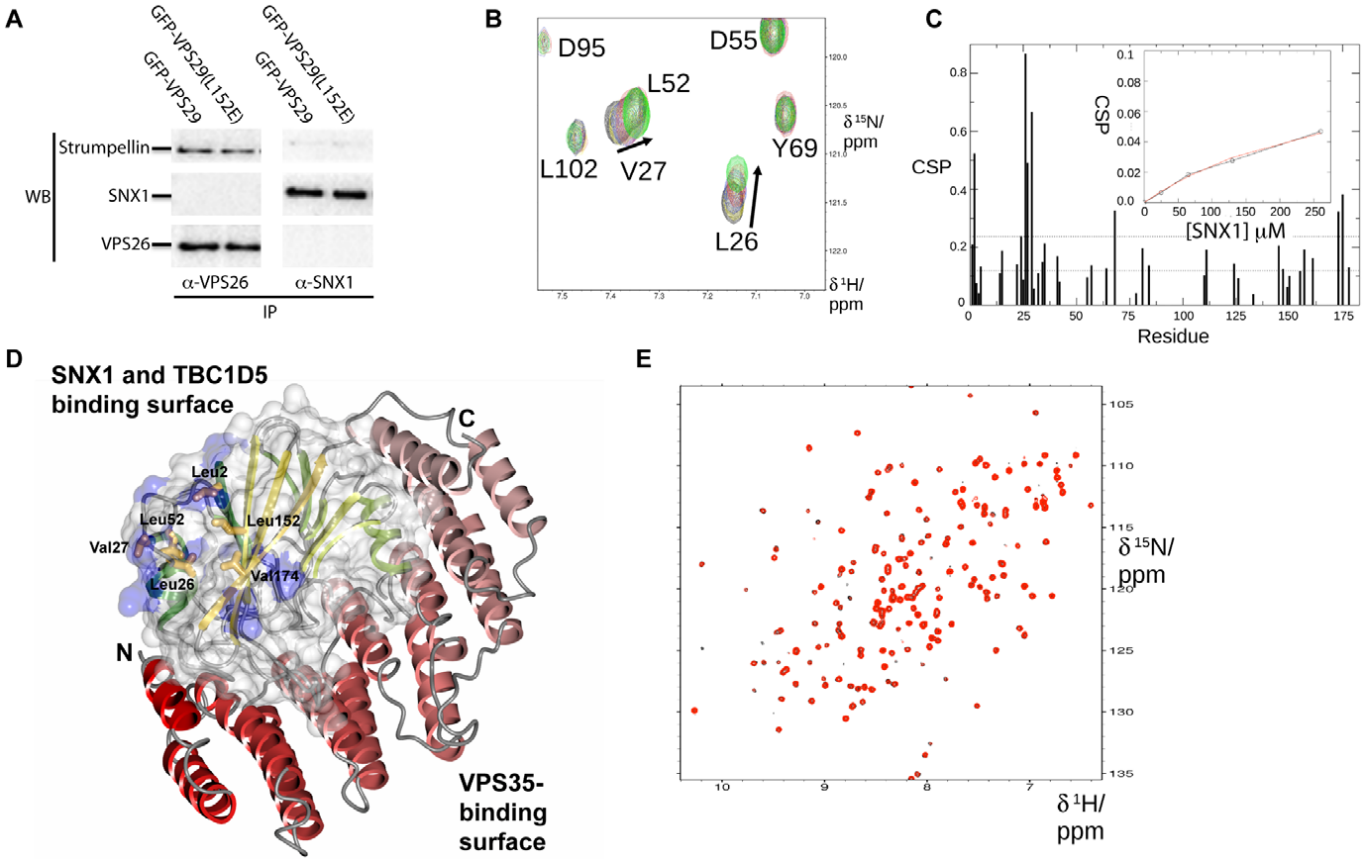
VPS29 binds specifically to SNX1 but with low affinity *in vitro*. (**A**) Immunoprecipitations from HeLa cells do not detect association of retromer with SNX1 even in the presence of increased levels of VPS29 expression. Cells expressing GFP-VPS29 or GFP-VPS29(L152E) mutant were subjected to immunoprecipitation with either VPS26 or SNX1 antibodies. VPS26 (and thus VPS29-containing retromer) associates readily with the effector complex containing strumpellin [32], confirming that known binding partners can be detected in the immuno-isolates. However, no SNX1 is detected, and furthermore, in reverse experiments SNX1 does not precipitate retromer indicating that their association *in vivo* is relatively weak or transient. (**B**) Titration of VPS29 with SNX1 in NMR experiments reveals specific but weak association *in vitro*. A selected region is shown for the ^15^N-HSQC spectra of VPS29 in the presence of increasing concentrations of SNX1. (**C**) Chemical shift perturbations are shown for VPS29 in the presence of SNX1. Inset shows a plot of the chemical shift perturbation for Leu26 NH as a function of SNX1 concentration. (**D**) SNX1 binds to VPS29 via the conserved hydrophobic surface on the opposite face to the metal-binding pocket and VPS35 binding interface. Residues that show the largest perturbations on SNX1 binding (>2 standard deviations) are mapped on the VPS29 structure in blue. The structure of VPS29 (surface, and green ribbons) is shown in complex with VPS35(476–780) (red ribbons) [24]. The side-chains of the VPS29 hydrophobic surface are indicated. (**E**) Mutation of the hydrophobic surface of VPS29 (L152E) prevents VPS29-SNX1 association. The [^1^H,^15^N]-HSQC spectra for VPS29(L152E) in the absence (black) and presence (red) of SNX1 indicates no significant association is occurring.

The final question we have addressed using NMR spectroscopy, is the potential interaction of VPS29 with associated regulatory proteins. Apart from VPS35, VPS29 has also been shown to interact with regulatory proteins; the SNX complex Vps5p/Vps17p in yeast [27], and the Rab GAP TBC1D5 in human cells [32]. Contrasting results have been seen for the binding of either TBC1D5 or the SNX complexes in mammalian cells. TBC1D5 interaction is readily detected in immunoprecipitations of GFP-tagged VPS29 and in yeast 2-hybrid assays, along with other effectors such as the WASH1/strumpellin complex [32] (R. Teasdale, personal communication). The interaction of VPS29 with mammalian SNX proteins is more contentious. To date, we have been unable to detect interactions of VPS29 or the retromer complex with mammalian SNX proteins in similar conditions as those used for TBC1D5 [27], [32]. For example, in experiments shown in **Fig. 8A**, we over-expressed GFP-VPS29 in HeLa cells in order to enhance potential complex formation, and performed immunoprecipitations with either the core retromer subunit VPS26 or the sorting nexin SNX1. As an initial control we first showed that VPS26 (and hence VPS29-containing retromer) readily binds the protein strumpellin as shown previously [32]. However, no SNX1 is detected in these immunoprecipitations, nor is retromer binding when SNX1 is used as bait, indicating a weak or transient interaction *in vivo*. The lack of binding is not due to competition by the antibody itself as experiments with epitope-tagged proteins have yielded similar negative results [27], [32]. Although other groups have detected interactions of retromer (both VPS35 and VPS29 subunits) with SNX proteins using ultra-sensitive yeast 2-hybrid assays, or over-expression of all retromer and SNX subunits together [20], [43], [44], there have been no reports of binding between endogenous mammalian proteins.

Overall the data suggests that mammalian SNX proteins either do not specifically associate with VPS29 or do so with much lower affinity than TBC1D5. To test this hypothesis we used NMR chemical shift titration experiments to assess the potential formation of the ∼140 kDa VPS29-SNX1 complex and determine if the two proteins can associate *in vitro* (**Fig. 8** **)**. Unfortunately, poor solubility of TBC1D5 constructs to date have precluded similar NMR experiments with this molecule. As shown in **Fig. 8B** and **8C**, clear chemical shift perturbations for VPS29 are observed on SNX1 addition, indicating specific interaction. Fortunately for the purpose of detection by NMR, the binding is indeed weak as judged by the observation of fast exchange and tracking of resonances in the ^15^N-HSQC spectra of VPS29 recorded in the presence of increasing concentrations of SNX1. Specific changes in a number of backbone resonances were observed prior to complete spectral broadening as the equilibrium shifts to a larger multimer. It was possible to plot the chemical shift perturbation for Leu26 on increasing SNX1 concentration to derive an approximate binding affinity (*K*_d_)>150 µM (**Fig. 8C**). Mapping of the shifted perturbations revealed that the primary site of SNX1 association occurs at the same hydrophobic surface identified in the yeast Vps29p protein as being important for Vps5p/Vps17p association, and for mammalian VPS29 binding to TBC1D5 (**Fig. 8D**) [27], [32]. In addition, alteration of this hydrophobic surface by mutating Leu152 to Glu abolishes the interaction (**Fig. 8E**). This mutation also blocks TBC1D5 binding [32], and is analogous to the L252E mutation in yeast Vps29p that abolishes association of retromer with Vps5p/Vps17p sorting nexins [27]. This provides compelling evidence that mammalian VPS29 can associate with both SNX proteins and TBC1D5 via an overlapping and conserved interface, albeit with a low binding affinity for SNX proteins *in vitro*.

## Discussion

In this work we have used a combination of techniques to examine the conformational changes in the VPS29 subunit of retromer between its apo and complexed state, and the functionality of different molecular interactions including metal binding and association with sorting nexin proteins.

### Metal binding and phosphatase activity of VPS29

With the discovery that VPS29 displays structural homology with metal-dependent PPP phosphatases, initial expectations were that retromer may regulate endosomal protein trafficking by dephosphorylating either cargo receptors, regulatory molecules or possibly even membrane lipids [27], [30]. We have carried out an analysis of the metal binding characteristics of VPS29 and find that although the protein is able to bind metals in the expected coordination geometry both in crystals and in solution, the association is of low affinity. Although metal binding by VPS29 in the cell cannot be totally excluded based solely on our *in vitro* studies, exhaustive phosphatase assays using either small molecule substrates [27] or a phosphorylated peptide derived from the retromer cargo molecule CI-MPR have so far failed to detect any significant enzymatic activity (this study and [24]). Although phosphatase activity of VPS29 was detected against the CI-MPR peptide previously [31], we note that protein samples used were significantly less pure than those used here and by Hierro *et al.* [24]. The activity was not quantified and was presumably quite low based on the fact that assays required very long incubation periods for detection of free phosphate. It is likely that this low level of activity may be due to impurities in the protein samples used.

In summary the weight of biochemical and structural evidence strongly suggests that neither VPS29 alone or the VPS29-containing retromer complex is an active phosphatase *in vitro*. Could retromer still be a functional phosphatase *in vivo*? Although it appears to be unlikely, the answer to this question still remains to be settled definitively. What is clear is that for activity to be present there must be processes occurring in a cellular context that are not recapitulated *in vitro*. At least two events would need to occur for enzymatic function. Firstly, a conformational change in the VPS29-VPS35 interface must take place to allow substrate access into the active site. This does not appear to happen *in vitro* but it is possible that in the cell, in particular in the context of the multi-component assembly involving SNX proteins, TBC1D5, lipid association, Rab7 and other molecules including cargo receptors, the VPS29-VPS35 interface may be altered. Secondly, previous analyses indicate that VPS29 lacks important active site residues found in related enzymes [27]. Therefore, it is probable that activity will depend on a co-factor protein. This could potentially involve VPS35, but again from our *in vitro* studies and other work [24] VPS35 alone is not sufficient to promote catalysis. It will be important to assess in a cellular environment whether depletion or mutagenesis of retromer affects dephosphorylation of potential target proteins before enzymatic activity of the complex can be ruled out entirely.

Based on differences observed in crystal structures of metal-bound and unbound VPS29 our modelling suggests metal binding might be able to regulate complex formation with the large subunit VPS35; however, we found no indication that metal binding by VPS29 affects retromer assembly. It is known that mutations in the metal-binding pocket cause the protein to be less stable, and to be produced at lower levels when expressed either in mammalian or bacterial cells [24], [27]. Is this instability a consequence of inhibited metal binding or simply a result of protein misfolding? Based on the fact that the wildtype protein can be isolated in high yields without bound metal when expressed in bacteria, and that NMR and ITC experiments indicate a low affinity for metal ions, we suggest that the latter is the more likely scenario. Therefore we propose VPS29 has a phosphatase-like fold, where residues that can coordinate metal cations have been conserved not for enzymatic activity but for purposes of protein stability, in particular within the context of the assembled retromer complex. We therefore propose that metal binding by VPS29 may not be required for its function.

### Dynamics of VPS29 structure in solution

NMR relaxation measurements are a unique resource for assessing protein dynamics on a residue-by-residue basis. Moreover these measurements, reporting ps through to ms motions, are complementary to the intimate structural details provided by X-ray structures, as often crystal contacts in the latter may artificially quench local motions [45]. There is a growing body of evidence to suggest that target sites of proteins have some degree of flexibility, which is intimately related to how the protein functions [46]. For example, in some cases relaxation measurements have uncovered important entropy switching, driving binding events [47], [48], [49]. From relaxation measurements on mouse VPS29, we have shown that, in the main, the protein secondary structure elements are relatively rigid in solution. Combining our residual dipolar coupling calculations, we propose that the α3 helix functions as a rigid body, which is presented to VPS35 in a pre-organized and correct orientation for assembly with respect to the main VPS29 protein scaffold and the metal binding face. Other interfacial loops nevertheless show some degree of flexibility, often paralleled with missing density in X-ray structures. It remains to be seen how the dynamic landscape of VPS29 changes upon complexation with VPS35 and its other target proteins.

### Scaffolding function of VPS29

We previously reported that a conserved hydrophobic surface on yeast Vps29p was critical for binding of the core retromer complex to yeast SNX proteins Vps5p and Vps17p [27], and the mammalian Rab GAP TBC1D5 [32]. Yeast two-hybrid studies also suggested that mammalian VPS29 is able to associate weakly with SNX1 and SNX2, both close homologues of yeast Vps5p, although it remains possible that this could be due to indirect association of mammalian VPS29 with endogenous yeast retromer subunits [20]. In this study we have addressed the question of whether mammalian VPS29 performs a similar role to yeast Vps29p, via direct interaction with SNX proteins. Using NMR spectroscopy to measure the binding of VPS29 to mammalian SNX1 we found a direct association between the two proteins. This association occurrs via the conserved hydrophobic surface, centred on residues Leu2, Leu52, Leu152 and Val174 on the opposite face to the metal-binding pocket and VPS35-binding interface. This has clear implications for retromer assembly with SNX proteins *in vivo*, whereby the SNX membrane tubulating scaffold binds not only to VPS29 as shown here but also to VPS35 as identified in yeast two-hybrid assays [50]. As this interface is also responsible for binding to TBC1D5, it will be important to determine firstly if mutation of the hydrophobic surface of VPS29 results in functional defects in retromer-mediated protein trafficking as expected, and if so how interaction with SNX proteins and TBC1D5 at this site are spatiotemporally coordinated in the cell. *In vitro* the direct association between VPS29 and SNX1 is of low affinity (*K*_d_>150 µM). *In vivo* we propose that the avidity of the core retromer and SNX interaction will be greatly increased by a number of factors, including oligomerisation of both complexes, lipid-protein interactions and coordinated binding of cargo and other membrane-associated proteins such as Rab7 [5], [19], [20], [51].

The region of both mammalian SNX1 and yeast Vps5p that associates with the core retromer complex has been narrowed down to an N-terminal domain predicted to contain little secondary structure [26], [52]. Interestingly, this region of SNX1 and SNX2 has been shown to be subject to phosphorylation, suggesting a potential role for post-translational modification in regulation of retromer/SNX interactions [53], [54]. We have previously suggested that retromer may bind to SNX proteins in a manner somewhat analogous to the interaction of linear nuclear localisation signals with the helical repeats of the importin proteins [2]. The exact contributions of the large VPS35 subunit and VPS29 to binding of SNX-dimers, and how these interactions fit within the model proposed by Hierro *et al.* [24] for retromer and SNX protein assembly onto endosomal membranes to form transport tubules remains an important question.

In conclusion we have provided evidence that although VPS29 retains metal-binding properties, it is not an active phosphatase enzyme *in vitro* and likely does not require metal binding for its function. NMR studies have allowed us to demonstrate conformational rigidity in the α3 helix of VPS29 that associates with VPS35, and have identified a conserved hydrophobic surface required for association of VPS29 with sorting nexins and other regulatory proteins. More generally we find that NMR spectroscopy is a fast, accurate and sensitive tool that will be well suited to future studies of VPS29 structure and binding to ligands such as TBC1D5, and potentially for screens to identify molecules that interfere with VPS29 function as investigative tools.

## Methods

### Crystal structure determination

VPS29 was purified and crystallised as described previously [27], with the minor alteration that PEG3350 was substituted for PEG3000 in crystallisation solutions. We previously found that Mn^2+^ was able to bind in the VPS29 putative active site and in this study we examined the ability of Zn^2+^ to bind in a similar fashion by soaking crystals in cryo solution (20% glycerol in mother liquor) containing 2 mM ZnSO_4_ for 15 min prior to flash cooling in the cryostream at 100 K. Crystals of Mn^2+^-bound VPS29 were prepared essentially as described previously. Briefly, crystals of apo VPS29 were soaked in cryo solution containing 50 mM MnSO_4_ for 30 min before flash cooling. Data was collected on the high-throughput protein crystallography beamline MX2 of the Australian Synchrotron. Data was integrated using XDS [55], scaled with SCALA [56], and model refinement and building was performed with PHENIX [57] and COOT [58]. For the low-resolution Zn^2+^-bound structure NCS restraints and group B-factors (one per residue) were applied during refinement. All structure images were made with CCP4mg [59].

### Isothermal titration calorimetry

VPS29 and VPS35 were purified essentially as described previously [27] except that 10 mM EDTA was included during lysis and affinity chromatography prior to gel filtration and buffer exchange into 20 mM HEPES (pH 7.4), 100 mM NaCl (ITC buffer). Experiments were performed using a Microcal iTC200 instrument at 298 K. For metal titrations metal chloride salts at 2.5 mM were titrated into 40 µM VPS29 in 13×3.1 µl aliquots. For VPS35 titrations, 100 µM VPS29 was titrated into 10 µM VPS35 in 13×3.1 µl aliquots in the presence or absence of 0.5 mM MnCl_2_, or 2.5 mM EDTA in both protein samples. Data was processed using ORIGIN to derive thermodynamic parameters Δ*H*, *K*_a_ (1/*K*_d_) and the stoichiometry *N*. Δ*G* and Δ*S* were derived from the relations Δ*G* = −RTln*K*_a_ and Δ*G* = Δ*H*−TΔ*S*.

### Phosphatase assay

Phosphatase activity against a phosphorylated peptide derived from the CI-MPR (CSSTKLVSFHDD(pS)DEDLLHI) were performed at room temperature in 20 mM HEPES (pH 7.4), 100 mM NaCl. VPS29 and VPS29-VPS35-VPS26 complexes were purified as described previously [25], [27]. Assays in a total volume of 50 µl used 23 µg peptide (diluted from a 2 mM stock solution) and either 0.25 mg calf intestinal alkaline phosphatase (CIAP) (New England Biolabs), 4 µg VPS29 or 10 µg VPS29-VPS35-VPS26 complex in the presence of 0.2 mM ZnCl_2_ or 1 mM MnCl_2_. Reactions were left for 2 h before addition of 100 µl Biomol Green reagent and incubation for 30 min, prior to measuring absorbance at 620 nm using a microplate reader. Released phosphate amounts (nmol.min^−1^.mg^−1^) were calibrated against a phosphate standard curve.

### Purification of SNX1 for NMR titrations

The human SNX1 gene was cloned into the pMCSG-GST vector by ligation-independent cloning for expression as a GST-fusion protein. Protein was expressed in Rosetta BL21(DE3)/pLysS *E. coli* cells and cells were lysed using a cell disruptor. After affinity purification on glutathione sepharose, GST-SNX1 was cleaved using Tobacco Etch Virus (TEV) protease overnight, and the resulting SNX1 protein further purified by gel filtration into NMR buffer, 10 mM HEPES (pH 7.5), 100 mM NaCl and 10 mM DTT.

### NMR spectroscopy

Isotopically labelled protein samples for NMR spectroscopy were prepared using the method of Marley *et al.* [60]. Briefly, a 2 l culture of BL21(DE3)-CodonPlus-RIL *Eschericia coli* cells expressing GST-VPS29 was pelleted, washed and resuspended in 500 ml M9 minimal media containing ^15^N-labelled NH_4_Cl_2_ or ^15^N-labelled NH_4_Cl_2_ and ^13^C-labelled glucose as required. Cells were induced with 0.8 mM IPTG and grown overnight at 293 K. Cells were lysed by French Press and protein purified by glutathione-sepharose affinity chromatography in 10 mM Tris (pH 8.0), 100 mM NaCl, 1 mM DTT, before cleavage with thrombin overnight at 293 K while bound to the column. Proteins were further purified by gel filtration chromatography in 10 mM HEPES (pH 7.5), 100 mM NaCl and 0.2 mM DTT. As the protein precipitated rapidly in air, 10 mM DTT was added to ^15^N/^13^C labelled sample for backbone assignments and the protein sealed under nitrogen in the NMR tube. In this manner VPS29 was stable for several weeks. This protein has an additional N-terminal GSPEFGTRDR sequence derived from the vector.

All NMR experiments were recorded at 298 K on a Varian Inova 600 MHz NMR spectrometer equipped with a cryoprobe and Z axis gradients. Triple resonance assignments were performed using ^15^N/^13^C labelled protein at a concentration of 0.5 mM in 90%/10% H_2_O/D_2_O. Backbone assignments were obtained by recording the following triple resonance three dimensional experiments; HNCO, HNCA, HN(CO)CA, HNCACB, CBCA(CO)NH and H(CBCA(CO)NH that was optimised for detection of Hα [61]. Assignments were further confirmed using a 3D ^15^N edited NOESY experiment recorded with a mixing time of 120 ms [62]. Titrations were performed by titrating ligands into 0.1 mM protein samples and recording a soFast ^15^N HMQC experiment [63].

All spectra were processed using nmrPipe [64] and analysed with XEASY [65]. ^1^D_NH_ RDCs were measured by using 0.1 mM ^15^N labelled VPS29 in the presence of an anisotropic media comprising 5% (wt/vol) C12E6/hexanol [66]. RDCs were obtained by comparing coupled spectra in the presence of the orienting media against spectra in the isotropic state by recording a 2D ^15^N IPAP-HSQC spectrum [67]. RDCs were measured using SPARKY [68]. ^1^D_NH_ RDCs were fitted to the X-ray structure of the mouse (PDB 1Z2X) or the human (PDB 2R17; PDB 1W24) VPS29 using the “Best fit” flag within PALES [41] and incorporated into the program as the isotropic (J) – aligned (J+^1^D_NH_) values. The ^1^D_NH_ RDCs were removed for couplings derived from severely overlapping peaks in the 2D IPAP spectra and mobile residues as inferred from ^15^N relaxation data. The error in the RDC was conservatively estimated as +/− 2 Hz, according to the ratio of the linewidth to the signal to noise.

^15^N Relaxation data was recorded on a ∼0.5 mM ^15^N/^13^C labelled VPS29 sample. ^15^N heteronuclear NOE spectra were recorded using TROSY type selection and with watergate suppression [69] owing to superior sensitivity compared to the sensitivity enhanced version [70] on the Varian cryoprobe. Three seconds of weak presaturation was used to generate the desired heteronuclear NOE and was applied on or off resonance at the amide proton frequency. T1 and T2 relaxation data was acquired as described using gradients for selection and for sensitivity enhancement [70]. The relaxation delay was sampled at 10, 50, 70, 100, 200, 300, 500 ms and 10, 30, 50, 70, 90, 110 ms for longitudinal and transverse relaxation measurements respectively. Peak intensities and relaxation times were measured using SPARKY and Monte Carlo simulation was used for error analysis in T1 and T2 times. The heteronuclear NOE was calculated and errors estimated from the base plane noise as implemented within the program relax [71].

### Immunoprecipitation experiments

VPS26, and SNX1 antibodies were described previously [11], and strumpellin antibody was from Santa Cruz (USA). HeLa cells stably transfected with VPS29-GFP or VPS29(L152E)-GFP constructs [27] were grown to ∼90% confluency in a 90 mm tissue culture dish. After removal of cell culture media, cells were washed with 5 ml of ice cold phosphate buffered saline (PBS). PBS was removed and cells were suspended in lysis buffer (20 mM HEPES-KOH (pH 7.0), 50 mM K-acetate, 2 mM EDTA, 0.1% triton and 200 mM sorbitol) and transferred to a 1.5 ml microtube. This is the same buffer used for successful native immunoprecipitation of yeast retromer complexes and SNX proteins [52]. The lysate was centrifuged at 10,000×g at 4°C for 5 mins and the supernatant transferred to a fresh tube containing 50 µl of protein-A sepharose (25% slurry). The lysates were precleared with protein-A sepharose for 30 mins at 4°C, followed by centrifugation at 10,000×g for 5 min, and the supernatant transferred to a fresh tube. Affinity purified polyclonal anti-VPS26 or anti-SNX1 antisera was added to each tube. The lysates were incubated at 4°C for 90 min on a rotating wheel after which 50 µl of protein-A sepharose was added and the lysates incubated for a further 60 min. After 4 washes with lysis buffer, the protein-A sepharose was dried in a vacuum concentrator, resuspended in SDS-PAGE buffer, heated to 95°C for 5 min and centrifuged to pellet the sepharose. Samples were analysed by SDS-PAGE and western blotting using antibodies against VPS26, SNX1 and strumpellin. Bound antibodies were detected using iodinated protein-A.

### Accession Numbers

Coordinates and structure factors for Mn^2+^ and Zn^2+^ bound VPS29 have been deposited in the RCSB PDB with IDs 3PSN and 3PSO respectively. Raw diffraction data is available on the Diffraction Images Repository (DIMER) http://xr-diffraction.imb.uq.edu.au.
